# A hybrid model of complexity estimation: Evidence from Russian legal texts

**DOI:** 10.3389/frai.2022.1008530

**Published:** 2022-10-31

**Authors:** Olga Blinova, Nikita Tarasov

**Affiliations:** ^1^Department of General Linguistics, Saint Petersburg State University, Saint Petersburg, Russia; ^2^Department of Philology, School of Arts and Humanities, HSE University, Saint Petersburg, Russia; ^3^Department of Technology of Programming, Saint Petersburg State University, Saint Petersburg, Russia

**Keywords:** complexity estimation, Russian legal texts, hybrid model, feature extraction, language model, transfer learning

## Abstract

This article proposes a hybrid model for the estimation of the complexity of legal documents in Russian. The model consists of two main modules: linguistic feature extractor and a transformer-based neural encoder. The set of linguistic metrics includes both non-specific metrics traditionally used to predict complexity, as well as style-specific metrics developed in order to deal with the peculiarities of official texts. The model was trained on a dataset constructed from text sequences from Russian textbooks. Training data were collected on either subjects related to the topic of legal documents such as Jurisprudence, Economics, Social Sciences, or subjects characterized by the use of general languages such as Literature, History, and Culturology. The final set of materials used contain 48 thousand selected text blocks having various subjects and level-of-complexity identifiers. We have tested the baseline fine-tuned BERT model, models trained on linguistic features, and models trained on features in combination with BERT predictions. The scores show that a hybrid approach to complexity estimation can provide high-quality results in terms of different metrics. The model has been tested on three sets of legal documents.

## 1. Introduction

The article focuses on a model for assessing the complexity of Russian legal texts. We describe the creation of a hybrid complexity estimation model involving 130 metrics combined with neural network encodings. Linguistic features take into account lexical, semantic, and syntactic properties of a text, its coherence, as well as sequences of part-of-speech tags, some word-formation patterns, and general-language frequency of lemmas. In addition, in-text references to other legal documents are considered (which is especially important when analyzing the laws).

The use of metrics in conjunction with efficient language coding allows one to estimate complexity from both linguistic parameters and implicit properties. The study (Deutsch et al., 2020) showed the success of such an approach in its most basic variation, i.e., adding neural network coding as a separate parameter for complexity estimation.

In terms of complexity, linguistic studies compare languages and dialects; language registers (or styles), and certain units (most notably words and sentences). The distinction between so-called “global” and “local” complexity is used (Szmrecsanyi and Kortmann, 2012): the first branch of studies is interested in exploring languages “as such”; the second one measures complexity in particular linguistic subdomains and deals with phonological, morphological, syntactic, semantic, lexical, and pragmatic complexity. The interlanguage comparison is dealt with by typologists (Dahl, 1993; Nichols, 2009), sociolinguists, and contactologists (McWhorter, 2001; Trudgill, 2011). Perceptual complexity is studied by psycholinguists (see e.g., Frazier, 1985). Computational linguists are also involved in complexity research, for an overview of approaches, see, for example, Collins-Thompson (2014). There is a rather long tradition of applying complexity assessment methods to Russian texts, for an overview, see e.g., Reynolds (2016) and Solnyshkina et al. (2022).

The interest in the complexity of legal language is quite natural. Lingua Legis has long been criticized for its verbosity, redundancy, lengthenings, syntactic overcomplication, archaic vocabulary, and unwarranted repetitions, see, e.g., Tiersma (1999) and Azuelos-Atias and Ye (2017).

A number of studies are aimed at highlighting the characteristics of legal documents that cause their difficulty, in developing approaches to the “Plain language movement,” and the composition of recommendations for “Plain writing.” Popular guides such as Wydick and Sloan (2019) give lawyers practical advice such as “omit surplus words,” “use verbs to express action,” “prefer the active voice,” “use short sentences,” etc. For the Russian research area, the problems associated with plain language have only been developed quite recently.

Russian legal texts have attracted the attention of complexity researchers, who, first, concentrated mainly on assessing legislative documents, and, second, used only readability formulas or other fairly simple and few measures.

For example, in Dmitrieva (2017), the texts of Constitutional Court decisions have been studied using a simple metric for assessing readability—the Flesch–Kincaid formula, adapted by Oborneva (2005). Saveliev and Kuchakov are also engaged in the study of complexity, see Kuchakov and Savel'ev (2018) and Savel'ev and Kuchakov (2019). In the cited articles, the authors have used only one lexical diversity measure (TTR, the value of which depends on the length of the text, hence the results of applying the metric may be questioned) and one syntactic measure (“Maximum Dependency Length,” the distance between the head and the dependent on the dependency tree, calculated as follows “for each particular text one value is taken which is the maximum for all sentences of the text”).

A new book (Knutov et al., 2020) on the complexity of legislative texts identifies nine factors, among them: “the share of verbs in the passive voice,” “the share of verbs in relation to the total number of words in the text,” “the average number of words in noun phrases,” “the average number of participial clauses located in sentences after the word being defined, per sentence,” “the average number of adverbial participle clauses per sentence,” “the average number of words in sentences,” “the average distance between dependent words in the sentence,” “the average number of roots per sentence,” and “the average number of words per paragraph.” Unfortunately, the authors Knutov et al. (2020) do not explicitly explain the reasons for their choice of parameters, which subsequently are not always clear to the reader. For example, it is not entirely clear what is meant by “the share of verbs in the passive voice,” probably only the share of passive participles (since grammemes of the voice on the morphological markup layer are not assigned to the finite forms of the verb).

Thus, the authors of the studies on the Russian legal language have focused on the complexity of legislative texts and the texts of judicial decisions. In addition, either only readability formulas or other, relatively few measures were used to estimate complexity.

We propose a complexity estimation model based on the combination of a variety of linguistic features and neural language model, trained on large-scale data and tested on three genre-diverse legal corpora. The goal of our research is to test different machine learning models trained on a set of linguistic features and compare them to the results achieved by the deep learning approach. We hypothesize that a hybrid approach has the potential to achieve better quality than any individual model by utilizing both the explicit encodings of complexity measures and implicit representations of the deep language model.

The remainder of this article is structured as follows: Section 2 provides a brief overview on the methods of automatic complexity estimation. Section 3 describes a training textbooks dataset and 3 corpora of legal documents in Russian used for testing the described model. Section 4 describes a set of linguistic features. Section 5 describes an encoding language model and introduces a training pipeline. Section 6 presents the experimental results. Section 7 concludes the article, outlines key contributions, and discusses the potential for future research.

## 2. Related works

Recent developments in the field of natural language processing have presented new possibilities for feature engineering, and introduced new supervised and unsupervised methods for complexity estimation. In general, modern approaches can be split into two distinct categories: traditional machine learning approaches and deep learning models.

Classical machine learning approaches typically utilize a set of specific engineered features in conjunction with a classification algorithm. The introduction of classification models has made it possible to outperform traditional readability scores, such as the Flesch–Kincaid using unigram features and naive Bayes classifier (Collins-Thompson and Callan, 2004). Later feature sets have been expanded to include more sophisticated lexical, grammatical, and discourse-based features (Feng et al., 2010). Xia et al. (2019) proposed a model for readability assessment for second language learners. The authors have utilized lexico-semantic features, parse tree features (such as grammatical relations), n-gram features, and discourse-based features. The results have shown the effectiveness of these features and the SVM classifier. Similar results can be found in the research articles by Szügyi et al. (2019) for texts in the German language and Santucci et al. (2020) where the authors achieved the best results for the Italian language using a set of linguistic features in conjunction with the Random Forest classifier. Lyashevskaya et al. (2021) showed the effectiveness of linguistic features for the task of complexity assessment of the texts written by Russian learners of English. Authors compared a random forest classifier, k-neighbors classifier, and logistic regression and concluded that a random forest classifier with TF-IDF vectors added as a feature obtains the best result. This result, in particular, shows the potential of combining the linguistic features and text encoding models.

Neural network-based approaches can be split into three general categories: general deep learning approaches such as feedforward neural networks-FNNs and convolutional neural networks-CNNs), recurrent-based networks-RNNs (including long short term memory-LSTM approaches; Staudemeyer and Morris, 2019) and transformer-based language models. Morozov et al. (2022) compared traditional machine algorithms with general deep learning approaches such as FNN and CNN. Neural network-based approaches outperformed traditional ones such as random forests in most tests. The authors carried out the experiments on three datasets in Russian, collected from textbooks. Sharoff (2022) proposes a method of linking neural predictions of text complexity to linguistic properties of data.

Additionally, some models utilize neural encodings as their document representations, instead of traditional linguistic features, n-gram encodings, or TF-IDF encodings. Word2vec (Mikolov et al., 2013), GloVe (Pennington et al., 2014), and FastText (Bojanowski et al., 2017) are known to provide generally high-quality encodings. Bosco et al. (2018) compare these encoding techniques in conjunction with RNN to evaluate complexity in the Italian language. These approaches, however, can be limiting in terms of application to a specific task. Transformer-based neural networks circumvent this issue by providing the opportunity to fine-tune the model to improve its effectiveness on a specific task. Mohtaj et al. (2022) discuss the applicability of the transformer-based BERT (Devlin et al., 2018) model for the task of readability assessment in German. Authors compare random forest regression with linguistic features, RNN-based model with baseline BERT encodings and fine-tuned BERT for regression. The results show the effectiveness of the fine-tuned BERT model.

Thus, previous studies demonstrate the potential of both linguistic features and BERT embeddings. Different research works show inconclusive results on the subject of model choice for complexity assessment tasks—random forests classification and regression, RNNs and FNNs, SVM models all show the potential to achieve high-quality results.

## 3. Data

Due to the lack of available supervised data on the topic of readability and complexity estimation in the Russian language for legal documents specifically, different datasets have been collected for the purposes of training and testing the model. Research on the complexity of Russian, in particular, commonly utilize textbooks data, see e.g., Dmitrieva et al. (2021). Thus, textbooks data are used for training to extract general patterns of text complexity for the language model. Additionally, this data has been used to train the final hybrid model and estimate its quality. For final testing, a set of legal documents has been used. These texts are used to test the effectiveness of the final model for the data, specifically related to the main task of this research—estimating the complexity of legal documents.

### 3.1. Training data

**Figure 1:**
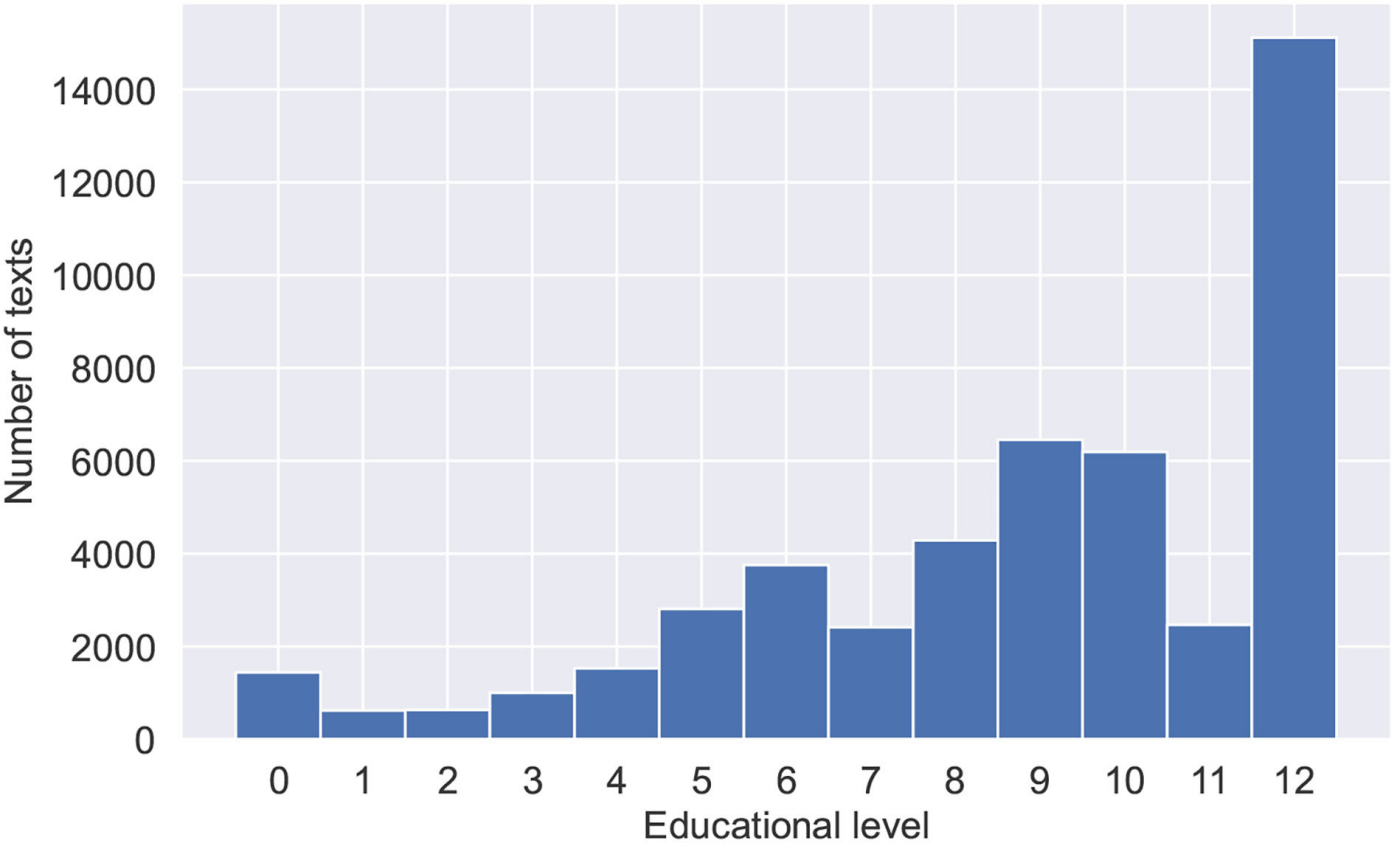
Distribution of texts across educational levels with 0 for texts from pre-school books, 1–12 for schoolbooks, and 12 for texts from university level books.

**Figure 2:**
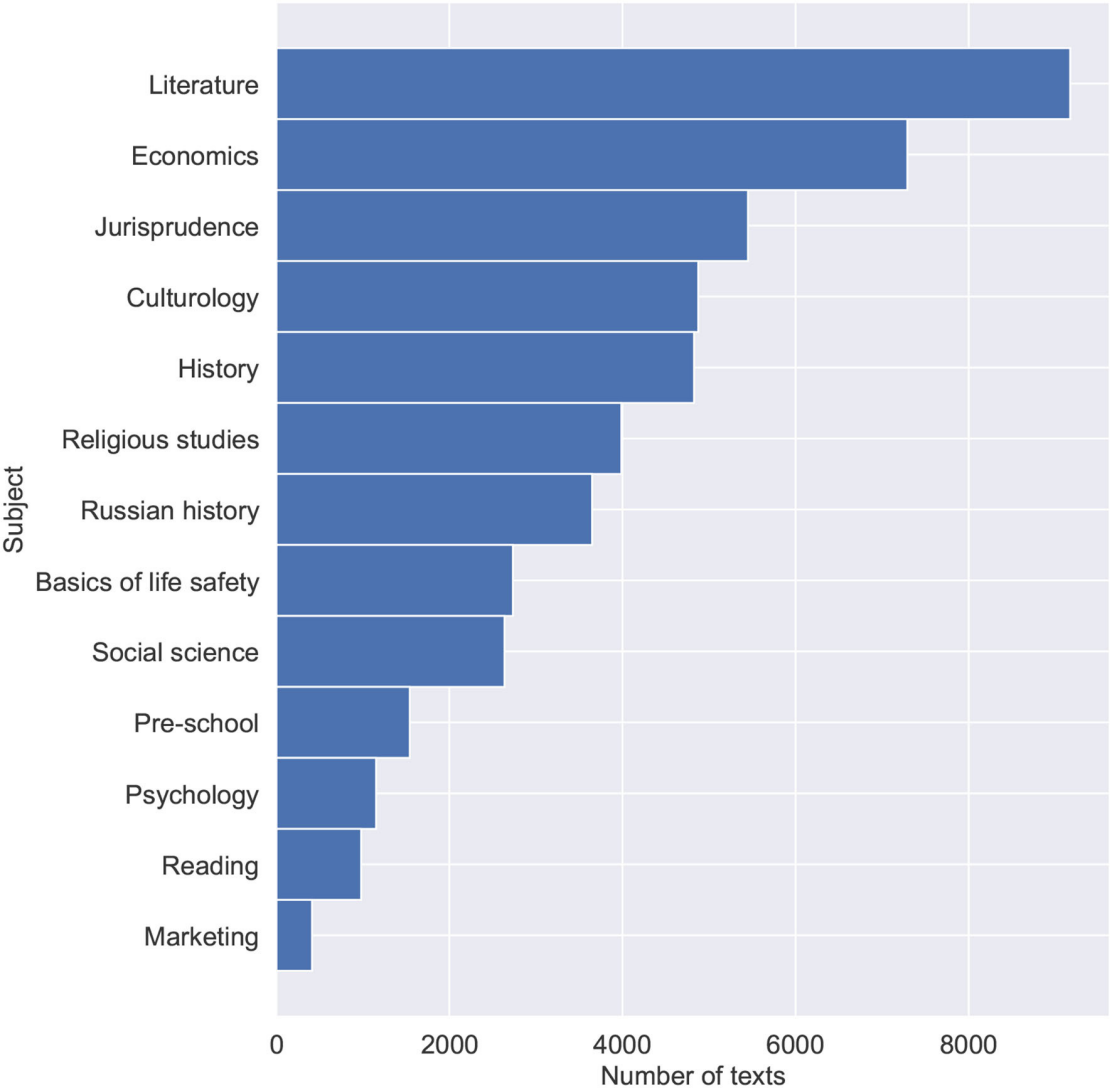
Distribution of texts across subjects.

Textbooks data were collected for the purposes of fine-tuning the Bert model and training the final hybrid model. The data consist of blocks of texts, randomly sampled from 1,448 textbooks in the Russian language. Textbooks were split into paragraphs to obtain a large volume of training data and provide a language model with shortened texts. Textblocks size limitation is important due to the fact that transformer-based language models have a maximum input sequence length typically ranging from 128 to 1,024 tokens. The data was also preprocessed, with tables of contents, additional ending information, and any non-textual information (tables, images, etc.) removed. Special symbols (excluding punctuation), occurring either naturally throughout the text or due to the errors of text file encodings were also removed. Training data were collected with variety and topicality in mind. Collected textbooks range in complexity from pre-school and elementary school to high school and university books. Table 1 shows statistical features of the training data. Figure 1 shows the number of texts for each educational level ranging from 0 for the pre-school level texts, 1–11 for years of school education, and 12 for university-level texts. Figure 2 shows the subjects and their corresponding amounts of texts.

**Table 1:** Statistics for the training data.

	**Total**	**Mean for each text block**	**Standard deviation**
Sentences	526,935	11	7
Tokens	9,939,730	204	151
Unique tokens	7,012,687	144	97

The subjects were chosen due to expected similarities with legal documents (i.e., the dataset includes textbooks on Jurisprudence, Social Sciences, and Economics) and as capable of presenting samples of texts in Russian with varying levels of complexity (i.e., the dataset includes textbooks on Literature, Culturology, and History).

### 3.2. Testing data

There is a significant number of Russian legal documents in the digital world; they are available, for example, through the legal information systems “ConsultantPlus” (Consultant Plus, 2022), “Garant” (Garant, 2022). This makes it possible to create extensive corpora.

The testing data are from three legal corpora. First, it is the “CorRIDA” corpus of Russian internal documents, consisting of 1,546 documents and containing 1,784 thousand tokens. Second, it is a corpus of decisions of the Constitutional Court of the Russian Federation “CorDec” of 3,427 thousand tokens, including 584 documents. Third, it is the “CorCodex” corpus of legislative documents, which contains 278 texts of codes, federal laws (a total of 3,227 thousand tokens).

Syntactic features are known to well predict textual complexity, see for example, Ivanov et al. (2018). Universal Dependencies (UDs) corpora have recently been increasingly used in assessing morphosyntactic complexity in both interlanguage comparison and comparison of text collections in the same language (Berdicevskis et al., 2018). Therefore, UDPipe was chosen as the basic markup tool. As a tool for morphological analysis, we used pymorphy2 (Korobov, 2015). When choosing a pre-trained UDPipe model, we relied on the accuracy statistics from CoNLL2018 (2018) and picked the “russian-syntagrus” model.

After preprocessing, automatic lemmatization, morphological markup, and syntactic parsing were performed. Each word form was assigned a double part-of-speech tag in terms of UDPipe and in terms of pymorphy2. The set of PoS tags of pymorphy2 allows, in particular, to distinguish between “ADJF” (full forms of adjectives), “ADJS” (short forms of adjectives), “VERB” (finite forms of the verb), “INFN” (infinitives), “PRTF” (full form of participles), “PRTS” (short form of participles), and “GRND” (adverbial participles). This is convenient for assessing complexity, in particular, because there is a positive correlation between the number of full adjectives (as well as participles and adverbial participles) and complexity and a negative correlation between the number of finite verbs and complexity, see Druzhkin (2016).

## 4. Linguistic features

To assess the complexity of Russian legal texts, 130 parameters were selected. The linguistic properties of Russian official texts (cf. the concept of “official-business style,” “rus. oficialno-delovoj stil”), described in research works on functional stylistics, as well as the features that are able to separate such texts from the texts of other styles when solving the problem of automatic classification by style, were taken into account.

All of the metrics used are conventionally divided into the following categories:

1. basic metrics,
2. readability formulas,
3. words of different part-of-speech classes,
4. n-grams of part-of-speech tags,
5. general-language frequency of lemmas,
6. word-formation patterns,
7. individual grammemes,
8. lexical and semantic features, multi-word expressions,
9. syntactic features,
10. cohesion assessments.

### 4.1. Basic metrics

The model provides the use of 28 basic metrics. Some of them are traditionally utilized in the tasks of classifying texts by complexity. All basic metrics can be divided into “basic quantitative” and “basic lexical” ones. The first ones are aimed, among other things, at taking into account the share of long words and long sentences (“long words” in the model are words consisting of four or more syllables). Basic lexical metrics implies calculating indexes of lexical diversity (simple TTR for word forms and lemmas; derived from TTR metrics “Yule's K” and “Yule's I,” whose values do not depend on text length), and calculating the shares of hapaxes (hapax legomena and hapax dislegomena).

### 4.2. Readability formulas

The use of readability formulas is a common method of complexity estimation. It is now utilized in combination with other methods, see, for example, (Benjamin, 2012), and is embedded in a variety of textometric resources. The described model uses five formulas: adapted Flesch–Kincaid formula (Solnyshkina et al., 2018), adapted Simple Measure of Gobbledygook (SMOG) formula, adapted formula for calculating the automated readability index ARI, Dale–Chale formula, Coleman–Liau index formula, see (Begtin, 2016). The formulas were adapted by Begtin using the text set which includes 68 documents categorized according to the educational level (from the third grade of elementary school to the sixth year of higher education).

### 4.3. Words of various part-of-speech classes

The metrics that take into account the shares of occurrences for words of various part-of-speech classes have been developed taking into account the differences between the markup tools used—UDPipe and pymorphy2, that is the differences between the sets of PoS tags (Straka and Straková, 2016) and (Korobov, 2015). Following Zhuravlev (1988), such indices were introduced into the model:

- “analyticity index” (the ratio of the number of function words to the total number of words);
- “verbality index” (the ratio of the number of verbs to the total number of words);
- “substantivity index” (the ratio of the number of nouns to the total number of words);
- “adjectivity index” (the ratio of the number of adjectives to the total number of words);
- “pronominality index” (the ratio of the number of pronouns to the total number of words);
- “autosemanticity index” (the ratio of the number of content words to the total number of words).

In addition, the ratio of the number of nouns to the number of verbs was used; the occurrences of short and full adjectives, and short and full participles are considered separately.

### 4.4. Part-of-speech n-grams

The information on n-grams of PoS tags was decided to involve for complexity analysis under the influence of studies on quantitative analysis of style (Antonova et al., 2011; Klyshinskij et al., 2013). In Antonova et al. (2011) the so-called “dynamic/static formula” was proposed to separate “dynamic texts” describing a sequence of events from the “static” ones containing descriptive passages, for more details see e.g., Dobrego and Petrova (2016). This metric allows one to successfully distinguish official documents (they are more “static”). The model described in this article uses 13 metrics of the category under discussion; for a complete list, the reader can address to https://www.plaindocument.org/.

### 4.5. General-language frequency

In assessing complexity, it is customary to take into account the length of the words of the text and their “familiarity” to the reader. The “familiarity” can be operationalized through the information on the general-language frequency of text lemmas. In the framework of our model for the accurate accounting of frequency data on the basis of large Russian corpora, a frequency list was created. This list contains about 1 million lemmas distributed into nine frequency bands using Zipf values, see about the method (Blinova et al., 2020b). Our complexity estimation model is able to calculate the proportion of lemmas belonging to each of the nine frequency bands and to distinguish between high-frequency, medium-frequency, and low-frequency lemmas.

### 4.6. Word-formation patterns

Derived words formed with the help of affixes are generally longer than generating ones. In addition, derivatives are more complex morphologically. This complexity makes derived words more perceptually difficult, which is confirmed experimentally, see Nagel' (2017). In our model, word-formation data are extracted from the level of lemmas, in each document the proportion of lemmas with endings of the type *cija, *nie, *vie, *tie, *ist, *izm, *ura, *ishhe, *stvo, *ost', *ovka, *ator, *itor, *tel', *l'nyj, *ovat' is calculated. This allows us to take into account the usage of deverbative and adjective-derived nouns, verb-derived adjectives and some derived verbs.

### 4.7. Grammemes

The model uses 17 metrics, taking into account, in particular: word forms in the genitive, instrumental, dative case, neuter nouns, third person verbs, full and short forms of passive participles, and finite verb forms with -*sja*.

### 4.8. Lexical and semantic features, multi-word expressions

The list of features assessed through a layer of lemmas or word forms is as follows

- the proportion of text-deictic expressions like *nastojashhij* “present,” *nizhesledujushhij* “following,” *vysheupomjanutyj* “aforementioned,” etc.,
- the proportion of graphic abbreviations,
- the proportion of letter abbreviations,
- the proportion of legal terms,
- the proportion of abstract lemmas,
- the proportion of lexical indicators of deontic possibility and necessity like *zapreshhat*' “to forbid,” *protivopravnyj* “wrongful,” *nadlezhashhij* “proper,” etc.,
- the proportion of multi-word prepositions like *v sootvetstvii s* “in conformance with,”
- the proportion of multi-word expressions used as a conjunction or conjunctive word like *vvidu togo chto* “due to the fact that,” *vsledstvie chego* “whereupon,”
- the proportion of light verb constructions like *okazyvat' sodejstvie* “to render assistance,” *osushhestvljat' podgotovku* “to conduct preparation,”
- the proportion of in-text references to the legislative acts, in particular, federal laws like *231-FZ* “Federal Law #31.”

To calculate the values of corresponding metrics, the set of user dictionaries is applied, that is, the value of the metric is calculated as the share of units that matched the unit from the dictionary. The dictionaries are available for download from https://www.plaindocument.org/.

### 4.9. Syntactic features

High syntactic complexity is a characteristic property of official texts. An extensive literature describes parameters for estimating sentence complexity, clausal complexity, and phrasal complexity. An up-to-date review is given in Kyle and Crossley (2018). An influential research in this field is Biber and Gray (2016). A large number of syntactic complexity measures have been used by Deutsch et al. (2020).

In the Russian language, the signs of complexity are considered to be, first of all, participial and adverbial participle clauses, complex, and compound sentences, see, for example, (Ljashevskaja, 1996; Ivanov et al., 2018). It is clear that the possibilities of syntactic complexity analysis are limited by the parsing format. Our model uses UDPipe for dependency parsing (see Section 3.1.2 above for details), utilizes 21 syntactic metrics, and takes into account, among other features: noun clause modifiers, adverbial clause modifiers, and various sentential complements, see https://www.plaindocument.org/ for details.

### 4.10. Cohesion

To assess referential cohesion, the measure “Cohes_1” (the number of noun repetitions in neighboring sentences) has been used. In addition, we have utilized the metric “Cohes_2,” which takes into account the number of repetitions of grammemes of tense and aspect for finite verbs (also in neighboring sentences).

At the end of the section, it is worth adding that some parameters of complexity estimation are not independent of each other, in particular, according to Zipf's law of abbreviation, word length correlates with word frequency, see for example, (Bentz and Ferrer-i Cancho, 2016). In addition, the representation in texts of the various features listed above can have both positive and negative correlations with the target complexity.

## 5. Experimental setup

**Figure 3:**
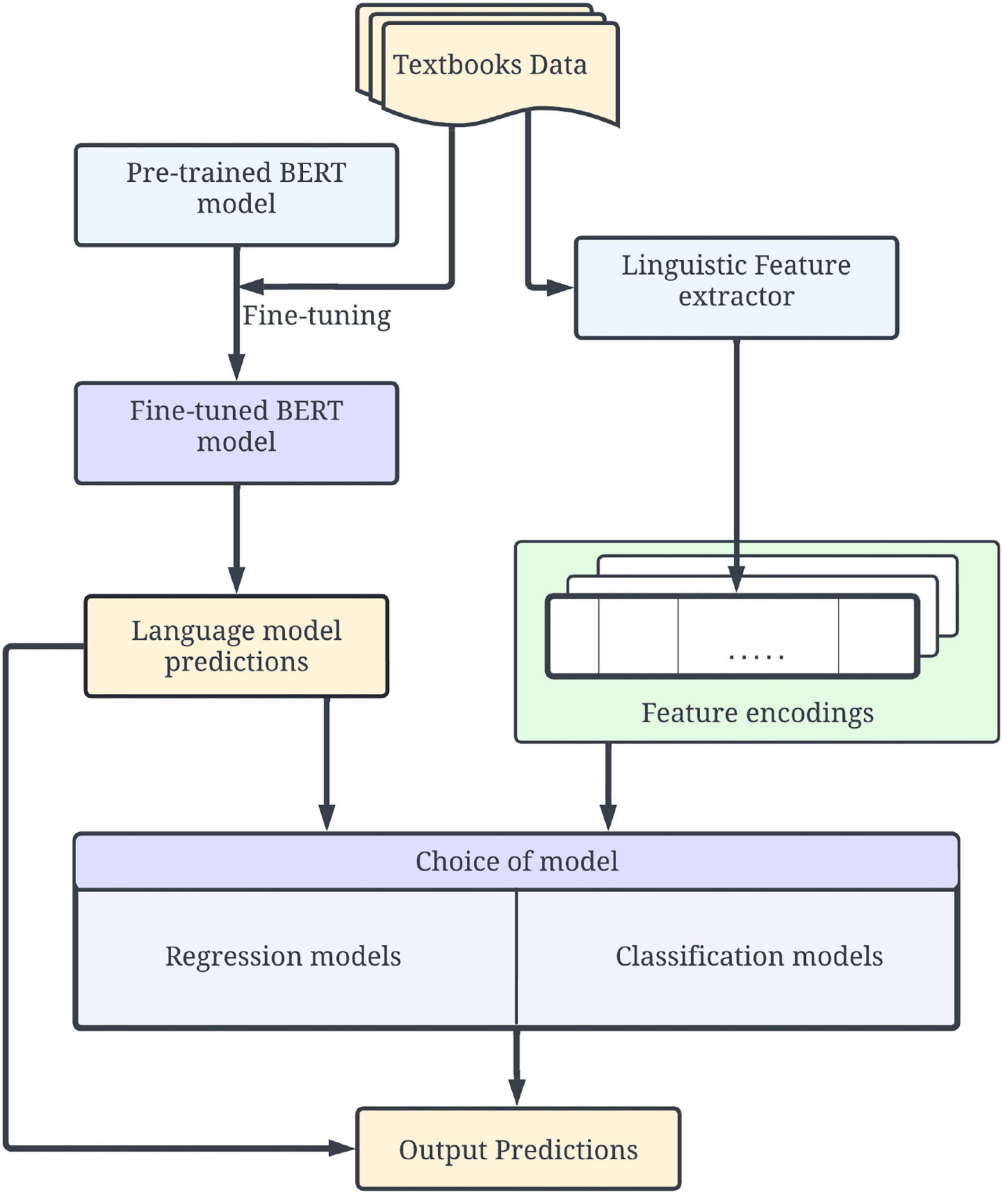
Proposed training and testing pipeline including three main modules: Language model, feature extractor, and final hybrid model. The final model outputs both the result of neural model and the final result of the hybrid model.

The resulting model consists of three main modules as shown in Figure 3. The training process is performed in two stages. In the first stage, a transformer-based BERT model is fine-tuned to obtain the initial complexity prediction for each text. The texts are additionally encoded using a set of metrics described in Section 4. Initial complexity predictions from the language model and feature encodings from predefined metrics are combined and propagated to the final testing module—a choice between different regression and classification models.

### 5.1. Language model predictions

Transformer architecture has been utilized for a number of different natural language processing tasks both as a standalone approach and as part of more complex combinational solutions. The basic idea of this approach lies in replacing recurrent layers with attention layers. This led to a significantly faster training process and better resource utilization due to parallelization capabilities, previously impossible for recurrent networks and LSTMs. As such, transformer is a fast and reliable method of language modeling that serves as a base for other more sophisticated and specialized algorithms. Bidirectional encoder representations from transformer—BERT model improves on this idea by introducing the bidirectional architecture, introducing transfer learning procedure. Since its inception, transfer learning has become an integral part of most text analysis solutions. This approach consists of two main steps, i.e., initial pre-training of the model on a large scale and universal set of tasks (next sentence prediction and masked language modeling for BERT) and the fine-tuning step designed to adapt the model for a specific task.

The method of fine-tuning transformer-based models pre-trained on large-scale data has been shown to provide high-quality text representations across different NLP tasks. This process is done by adding an additional linear layer at the end of the pre-trained model and training it for a few epochs. The intuition behind this approach is that the initial pre-trained model learns generic language patterns, while the fine-tuning process allows the model to learn task-specific patterns (Merchant et al., 2020).

In this research, we utilize a base version of RUBERT (Kuratov and Arkhipov, 2019), obtained from the Huggingface transformers library (Wolf et al., 2019). The model is pre-trained for the Russian language on the data obtained from various social media datasets. Initial pre-trained model consists of 12 layers, 768 hidden units per layer, and 12 attention heads.

Due to a large number of categories for complexity in our dataset and their ordered nature, we propose that the regression, approach could be more applicable. By defining the task as regression we can potentially achieve higher quality predictions in the corner cases. Whereas, classification predicts one of the outcomes without the context of their proximity to each other, the regression model can provide useful information by making predictions that lie closer to the real values even if not exact.

**Figure 4:**
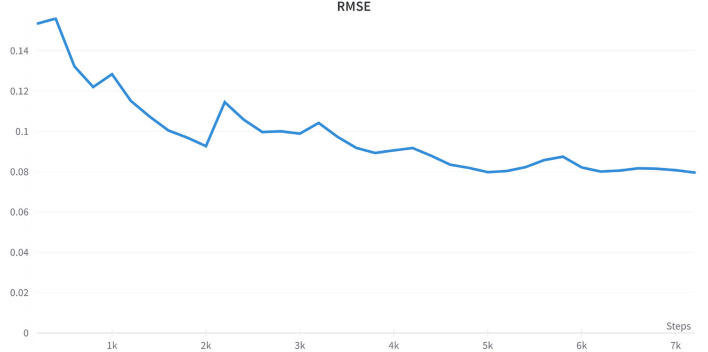
Quality improvement during fine-tuning of the language model indicated by the RMSE metric.

Our approach employs a standard fine-tuning process. It utilizes a pre-trained RUBERT tokenizer to split text blocks into tokens and add special padding and [CLS] tokens. Encodings are then passed through the model until the last layer where the hidden state of the [CLS] token is extracted and passed through a dense layer with a hyperbolic tangent activation function. For fine-tuning, we used AdamW optimizer (Loshchilov and Hutter, 2017) with a 2e-5 learning rate, 16 batch size, three epochs, and 1e-2 weight decay. The model is optimized to find the best result in terms of RMSE loss for validation subset of data – 10% of the initial texts. Figure 4 shows the improvement in quality during the fine-tuning process.

### 5.2. Combining approach

To combine the linguistic features with the language model, we obtain the output from the fine-tuned BERT model and use it as a feature in combination with linguistic features. This final vector representation is passed to another model. Deutsch et al. (2020) utilize an SVM classifier for their choice of the final model for its simplicity and frequent use in tasks involving adding numerical features.

In this research, we want to additionally evaluate the potential of other types of models, including regression. With a large number of complexity classes (there are 13 categories in our case), there is a potential that regression models can provide a better result due to their ability to obtain a complexity score rather than direct class prediction. This can improve the quality and usability of the model. Whereas classification model can confuse between any class during the inference, regression model errors will still be close to the target value.

We have tested the quality of six models: linear regression, XGBoost (Chen and Guestrin, 2016) for regression, FNN for regression, SVM for classification, random forest classification, and XGBoost for classification. Linear regression and SVM classifier have been chosen to provide a baseline quality estimation using simple approaches. SVM classifier is also the model commonly utilized for complexity estimation task. The regression FNN model is a dense neural model which, in our case, consists of three hidden layers, 128 hidden units each. The model has been trained with Adam optimizer with 1e-3 learning rate. Random forest is a commonly used ensemble approach that trains a number of weaker decision trees on subsets of data and combines them into a stronger predictor, reducing the over-fitting. Extreme Gradient Boosting or XGBoost is a gradient-boosted decision tree (GBDT) machine learning library. It uses a technique where new models are introduced to correct the errors made by existing models. We have tuned the hyperparameters for this algorithm using the Hyperopt library (Bergstra et al., 2013) to build 500 estimators for classification and regression tasks and find the set of optimal model parameters for each.

## 6. Experimental results

To compare the effectiveness of each method we use a set of metrics. Classification accuracy is measured as a basic percentage of correct predictions. For regression models, this and all future classification metrics are defined by rounding the predictions to the closest category. Accuracy for university-level texts (AUT) measures the accuracy of classification for texts with maximum complexity rating. It is measured to ensure the quality of predictions for texts of higher difficulty, presumably composing a large amount of legal texts data. Precision, recall, and f-measure are calculated using the weighted average of the values for each class. Root mean squared error is measured to find the difference between predictions and true values in the regression problems. Lower values indicate higher quality. For classification algorithms, the predictions are mapped to a 0 to 1 space. R2 score—coefficient of determination is a more straightforward regression score typically ranging from 0 to 1, however, can be arbitrarily worse. Table 2 shows the results of testing for each model.

**Table 2:** Testing results show the quality across different models and model combinations.

	**Accuracy**	**AUT**	**Precision**	**Recall**	**F1**	**RMSE**	**R2**
Fine-tuned BERT	0.6308	0.9502	0.6366	0.6308	0.6311	0.0762	0.9173
**Regression models**							
Linear Regression with features	0.2095	0.2793	0.3821	0.2095	0.2333	0.1985	0.4399
Linear Regression combined	0.7053	**0.9873**	0.7163	0.7053	0.7028	0.0621	0.9451
XGBoost with features	0.1491	0.2531	0.3871	0.1491	0.1378	0.2005	0.4283
XGBoost combined	0.5782	0.8055	0.6273	0.5782	0.5946	0.0728	0.9246
FNN with features	0.4918	0.8334	0.4834	0.4918	0.4839	0.1786	0.5465
FNN combined	0.7358	0.9741	0.7317	0.7358	0.7308	0.0654	0.9391
**Classification models**							
SVM with features	0.3738	0.9455	0.3161	0.3738	0.2731	0.3226	-0.4787
SVM combined	0.3741	0.9462	0.3162	0.3741	0.2732	0.3226	-0.479
Random Forests with features	0.6002	0.9422	0.5952	0.6002	0.573	0.2179	0.3252
Random Forests combined	0.7775	0.9814	0.7814	0.7775	0.7723	0.0863	0.894
XGBoost with features	0.6039	0.9137	0.5888	0.6039	0.5867	0.1968	0.4493
XGBoost combined	**0.7855**	0.9834	**0.7839**	**0.7855**	**0.7835**	**0.0605**	**0.9479**

Bold indicates the best result for each metric.

In all cases, the introduction of the BERT predictions provided an improvement in comparison with models trained only on linguistic features. In almost all cases, the results were improved over the baseline BERT predictions. As highlighted in the table, the XGBoost classification model trained on linguistic features and language model predictions achieved the best results on almost all metrics. This is true even for regression-based metrics, indicating that incorrect predictions were close to the real scores. For regression models, the introduction of the language model predictions provided a more significant improvement in quality with the highest quality being achieved by the three-layer neural network. A linear regression model with language model predictions achieved the best quality of predictions for university level text and obtained accurate predictions in general.

## 7. Discussion

The resulting model has been tested on the legal documents data. Initial predictions were obtained using the fine-tuned BERT model, combined with linguistic features and passed through the XGBoost model.

**Figure 5:**
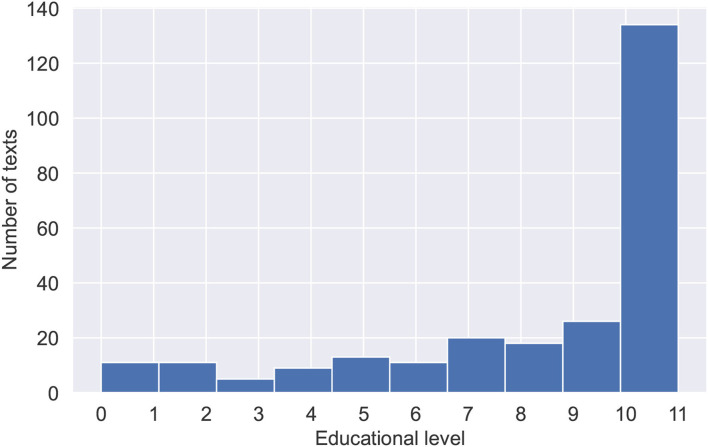
Complexity distribution for CorRIDA data, excluding the university-level texts.

For the “CorDec” dataset, all documents were identified to have the highest complexity. For the “CorCodex” data, 95% of documents were given the maximum complexity score. “CorRIDA” data were found to be the most diverse with 83% of data identified as highly complex documents. Figure 5 shows the distribution for the remaining files.

The observed differences between the three datasets are generally consistent with our expectations. The “CorRIDA” corpus of Russian internal documents and acts includes a little-studied category of legal texts, the so-called “internal documents.” They are created in a particular state organization and regulate only the activity of this organization. The corpus contains documents addressed to the “ordinary citizen”: to the applicant at the university, to a visitor at a museum or theater, to the patient at the clinic, etc. Apparently, it is primarily such official texts that we (i.e., Russian speakers who are not professional lawyers) periodically have some dealings with. For example, we sign “Consents to personal data processing,” “Informed consents to medical intervention,” or “Contracts for the provision of services.” The internal documents are not always written by lawyers, standard templates are used to form them, but most importantly they are addressed to “ordinary speakers.” Unsurprisingly, the “CorRIDA” dataset does not only consist of texts with maximum level of complexity.

The Constitutional Court Decisions, moreover, are written by highly professional lawyers, for a description see (Blinova et al., 2020a). Such documents nominally are addressed to a wide range of citizens. However, lawyers themselves are concerned about the excessive complexity of the language of Constitutional Court decisions. Thus, Dmitrieva (2017) concludes that “the average judgment of the Court is written in too complicated language, aimed at a reader with a postgraduate education.”

The third dataset (the “CorCodex” corpus) consists mainly of the texts of federal laws and codes. Complaints about the difficulty and incomprehensibility of the laws can be considered truisms, cf. the witty quote from Assy (2013): “complaints about the excessive complexity of the law are as old as the law itself.” Existing research works show that the complexity of legislative texts increases over the years, see (Kuchakov and Savel'ev, 2018). Indeed, according to our results, only 11 of the 278 “CorCodex” corpus texts did not receive a score other than the maximum one, while six documents belong to the period from 1993 to 1999, four were written in the period from 2000 to 2003, one text was draft in 2010.

## 8. Conclusion

In this article, we have proposed a method of complexity prediction model hybridization. We have collected a training dataset with texts from textbooks in Russian with various levels of complexity on the subjects either related to the field of Jurisprudence or providing general language characteristics. Our research demonstrates the effectiveness of the BERT deep language model by itself and in combination with predefined linguistic features. We have measured the quality of models on a set of metrics aimed to find the model, capable of high accuracy in general, high quality of predictions for complex texts in specific, and low distance between predicted and actual values even in case of errors. Our findings show that additional language model predictions provide a boost in quality for all regression and classification-based models. The XGBoost model with tuned parameters, trained on features and language model predictions, has obtained the best result on training data and has been used in the final testing step.

The additional tests on legal documents have shown the effectiveness of this approach in identifying complex texts, but have identified its biggest drawback, i.e., data dependence. The general model such as the one presented in this article was not able to capture small differences in complexity between texts which can be considered complex by default.

Future work involves collecting a supervised dataset, containing a number of Russian legal documents, labeled by complexity. Following a general workflow established in this research, we aim to create a powerful text complexity predictor for both the general usage and the legal domain. Meanwhile, the complexity assessment process established in this article is most suited toward expert analysis. It can, however, be adapted for broader usage to provide recommendations on drafting texts aimed at a wide range of users. In the context of legal documentation, this can be useful as a way to facilitate the communication between legal experts and the citizens as well as to simplify the work of legal drafters.

## Data availability statement

The original contributions presented in the study are included in the article/supplementary materials, further inquiries can be directed to the corresponding author.

## Author contributions

OB reviewed the literature, designed the set of features for text complexity assessment, participated in the formation of three legal corpora used as testing data, in the design of the experiment, and contributed to writing the article. NT designed the experiments, coded the algorithms, performed the computations and experiments, and contributed to writing the article. Both authors contributed to the article and approved the submitted version.

## Funding

The presented research was supported by the Russian Science Foundation, project #19-18-00525 Understanding official Russian: The legal and linguistic issues.

## Conflict of interest

The authors declare that the research was conducted in the absence of any commercial or financial relationships that could be construed as a potential conflict of interest.

The reviewer TP declared a shared affiliation with the authors at the time of the review.

## Publisher's note

All claims expressed in this article are solely those of the authors and do not necessarily represent those of their affiliated organizations, or those of the publisher, the editors and the reviewers. Any product that may be evaluated in this article, or claim that may be made by its manufacturer, is not guaranteed or endorsed by the publisher.
